# Inhibiting neuronal AC1 for treating anxiety and headache in the animal model of migraine

**DOI:** 10.1016/j.isci.2023.106790

**Published:** 2023-04-28

**Authors:** Ren-Hao Liu, Mingjie Zhang, Man Xue, Tao Wang, Jing-Shan Lu, Xu-Hui Li, Yu-Xin Chen, Kexin Fan, Wantong Shi, Si-Bo Zhou, Qi-Yu Chen, Li Kang, Qian Song, Shengyuan Yu, Min Zhuo

**Affiliations:** 1 Institute of Brain Research, Qingdao International Academician Park, Qingdao 266000, China; 2 Center for Neuron and Disease, Frontier Institutes of Science and Technology, Xi’an Jiaotong University, Xi’an 710049, China; 3 School of Biomedical Engineering, Shanghai Jiao Tong University, Shanghai 200240, China; 4 Department of Neurology, the First Medical Centre, Chinese PLA General Hospital, Beijing 100853, China; 5 Neuroscience Research Center, Institute of Mitochondrial Biology and Medicine, Key Laboratory of Biomedical Information Engineering of Ministry of Education, School of Life Science and Technology and Core Facilities Sharing Platform, Xi’an Jiaotong University, Xi’an 710049, China

**Keywords:** Psychiatry, Pharmacology, Molecular neuroscience

## Abstract

Migraines are a common medical condition. From a basic science point of view, the central mechanism for migraine and headache is largely unknown. In the present study, we demonstrate that cortical excitatory transmission is significantly enhanced in the anterior cingulate cortex (ACC)—a brain region which is critical for pain perception. Biochemical studies found that the phosphorylation levels of both the NMDA receptor GluN2B and AMPA receptor GluA1 were enhanced in ACC of migraine rats. Both the presynaptic release of glutamate and postsynaptic responses of AMPA receptors and NMDA receptors were enhanced. Synaptic long-term potentiation (LTP) was occluded. Furthermore, behavioral anxiety and nociceptive responses were increased, which were reversed by application of AC1 inhibitor NB001 within ACC. Our results provide strong evidence that cortical LTPs contribute to migraine-related pain and anxiety. Drugs that inhibit cortical excitation such as NB001 may serve as potential medicines for treating migraine in the future.

## Highlights

• Chronic migraine induces behavioral anxiety and periorbital hyperalgesia in rats
• Chronic migraine rats show enhanced synaptic excitatory transmission in ACC
• AC1-dependent plasticity contributes to the enhanced synaptic transmission in ACC
• Inhibiting AC1 reverses hyperalgesia and anxiety in chronic migraine rats

## Graphical abstract

**Figure:**
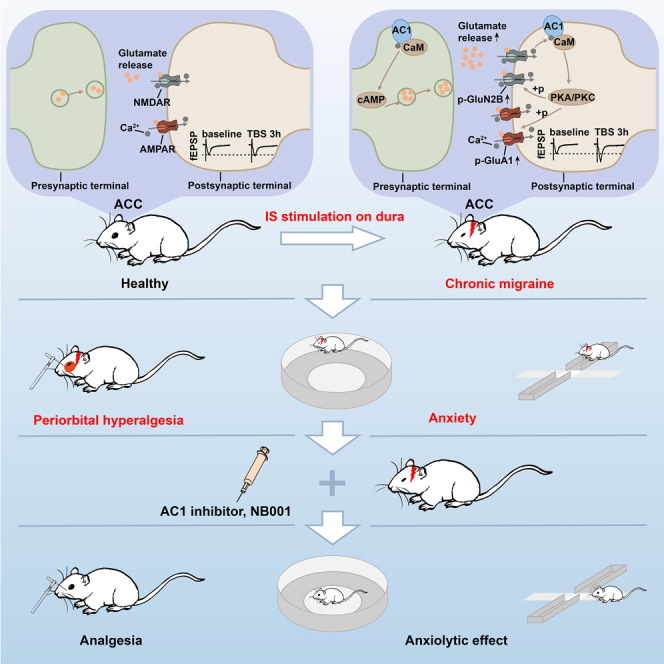

## Introduction

Migraine is a major form of chronic pain and it frequently has associated comorbidities such as somatosensory diffusible allodynia and anxiety. The central mechanism for migraine and its associated allodynia and anxiety remains unknown. By using an animal model of migraine (trigeminovascular models induced by inflammatory stimulation of dura), previous studies have demonstrated that the inflammation of the dura triggers the reduction of nociceptive thresholds and the enhancement of neuronal responses to both nociceptive and non-nociceptive stimuli.^1^^,^^2^^,^^3^^,^^4^^,^^5^ Most of these studies focused on the trigeminal ganglion (TG) and thalamus.^2^^,^^6^ For example, neurons in TG and posterior thalamus were sensitized and showed long-lasting hyperexcitability to innocuous and noxious stimulation of the paws in adult rats.^5^^,^^7^ Less is known about the possible roles of the anterior cingulate cortex (ACC), a key cortical region for pain perception and chronic pain.^8^^,^^9^^,^^10^ Recent human brain imaging studies showed that there was loss of the gray matter volume in the ACC of migraineurs.^11^^,^^12^^,^^13^ In addition, the impairment in glutamate uptake in mice with familial hemiplegic migraine promoted N-methyl-D-aspartate (NMDA) spike generation in ACC neurons and enhanced output firing of these neurons.^14^ These findings suggest that ACC excitatory transmission may play an important role in migraine, although the exact molecular mechanism remains uninvestigated.

ACC synapses are highly plastic.^8^^,^^9^ The changes of plasticity in the ACC (including the increase of presynaptic neurotransmitter release probability, the increase of the expression of postsynaptic α-amino-3-hydroxy-5-methyl-4-isoxazole-propionic acid (AMPA) receptors) can be triggered by different types of peripheral injuries, and further contribute to chronic pain and anxiety.^15^^,^^16^^,^^17^ A variety of signaling molecules are involved in the intracellular pathways of ACC plasticity, such as NMDA receptor, adenylyl cyclase 1 (AC1), protein kinase Mζ, and AMPA receptor.^8^^,^^18^ Among these targets, AC1 is thought to be a selective target for the treatment of chronic pain. Unlike the side effects such as ataxia and sedation of ion channel antagonists (such as NMDA receptors), AC1 knockout does not affect the key physiological functions such as learning process and cognitive function.^19^ These findings strongly indicate that AC1 may serve as a selective target for the treatment of chronic pain, although most of previous studies use animal models of somatosensory and visceral pain.

In the present study, we applied inflammatory mediators to the dura mater of rats to establish chronic migraine. We found that the phosphorylation of NMDA and AMPA receptors in the ACC was enhanced and the phosphorylation was similar to other forms of chronic pain. A selective AC1 inhibitor, NB001, which has recently proved to be safe in animals and humans, applied locally in the ACC or orally, produced powerful analgesic and anti-anxiety effects on chronic migraine rats. Our results strongly suggest that ACC plasticity regulates the chronic migraine and AC1 may be a potential target for future treatment of chronic migraine.

## Results

### The mechanical withdrawal hyperalgesia in migraine model rats

**Figure 1:**
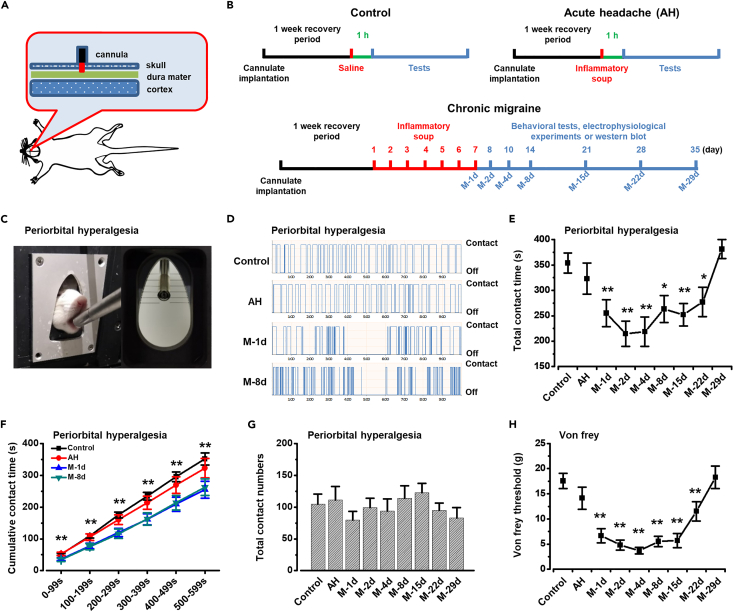
Facial hyperalgesia test in different migraine model rats (A) Schematic of cannulate implantation for saline or IS stimulation. (B) Schematic of different treatment groups. Control group (upper left) was tested at 1 h after saline stimulation. Acute headache (AH) group (upper right) was tested at 1 h after IS stimulation. Chronic migraine group (lower) was tested respectively on migraine first day (M-1d), second day (M-2d), fourth day (M-4d), eighth day (M-8d), 15th day (M-15d), 22nd day (M-22d), and 29th day (M-29d). (C) An example showed a rat that was drinking milk while its facial regions were contacting the stimulating filaments of mechanical stimulation module. (D) Four sample traces showed the automatic recordings of drinking behavior in a duration of 10 min of a control rat (upper), an AH rat (middle), an M-1d rat (middle), and an M-8d rat (lower). (E) Migraine (M-1d: n = 25, *t*_(50)_ = 3.015, p = 0.004; M-2d: n = 22, *t*_(47)_ = 4.471, p = 4.910E-05; M-4d: n = 17, *t*_(42)_ = 3.956, p = 2.875E-04; M-8d: n = 17, *t*_(42)_ = 2.761, p = 0.009; M-15d: n = 17, *t*_(42)_ = 3.319, p = 0.002; M-22d: n = 17, *t*_(42)_ = 2.255, p = 0.029; unpaired *t* test) rats showed significant lower contact time than that in the control rats (n = 27) and the significance lasted for about three weeks. AH (n = 22; *t*_(47)_ = 0.880, p = 0.383, unpaired *t* test) and M−29 rats (n = 11; *t* _(36)_ = −0.825, p = 0.415, unpaired *t* test) showed similar contact time to control rats. (F) Comparison of cumulative contact time over the test period of 10 min between 4 different groups (*F*_(2, 396)_ = 30.250, p = 5.948E-13, two-way ANOVA). (G) There was no difference in total contact numbers among these different groups. (H) AH rats showed no difference in periorbital threshold with control rats (Control: n = 20; AH: n = 18; *t*_(36)_ = 1.264, p = 0.214, unpaired *t* test). Migraine rats (M-1d: n = 18, *t*_(36)_ = 5.166, p = 9.027E-06; M-2d: n = 18, *t*_(36)_ = 6.744, p = 7.137E-08; M-4d: n = 17, *t*_(35)_ = 7.724, p = 4.560E-09; M-8d: n = 17, *t*_(35)_ = 6.162, p = 4.732E-07; M-15d: n = 17, *t*_(35)_ = 5.512, p = 3.396E-06; M-22d: n = 17, *t*_(35)_ = 3.287, p = 0.002; unpaired *t* test) showed decreased periorbital threshold for 3 weeks and returned on the 29th day (n = 11; *t*_(29)_ = −0.266, p = 0.792, unpaired *t* test). *∗*p *<* 0.05; *∗∗*p < 0.01 vs. control group. Data are represented as mean ± SEM.

Hyperalgesia and allodynia are two important symptoms of chronic pain. Previous studies have shown that the periorbital nociceptive threshold was reduced in migraine animals.^1^^,^^20^ To investigate the change of periorbital hyperalgesia in different headache rats, we performed improved orofacial stimulation test system and von Frey monofilaments on the periorbital regions of control, acute headache, and chronic migraine rats. The establishment process of animal model is shown in Figure 1B. Control (control group, Figure 1B upper left) or AH (acute headache (AH) group, Figure 1B upper right) group only received saline or inflammatory soup (IS) stimulation for one time, respectively. Chronic migraine group received IS stimulation for 7 days and one time per day (migraine (M) group, Figure 1B lower). Furthermore, in order to observe whether the migraine state is long-lasting, the migraine group rats were tested respectively on the first day (M-1d), second day (M-2d), fourth day (M-4d), eighth day (M-8d), 15th day (M-15d), 22nd day (M-22d), and 29th day (M-29d) of migraine (Figure 1B lower).

Figures 1C and 1D showed the testing process of improved orofacial stimulation test system which was similar to those in previous studies.^21^ When the rats drank milk, the stimulating filaments would attach to the periorbital regions of the rats and the duration of drinking was recorded until the rats left the drinking window. To avoid the potential influence of postoperative pain, we performed the orofacial operant test with stimulation modules 2 weeks after operation. During the 10-min testing period, the control and AH rats had similar cumulative contact time (Figure 1F). Meanwhile, there was no significant difference in total contact time between control and AH rats (Figure 1E). In addition, the total contact time of chronic migraine rats at different time points was also recorded. The total contact time of M-1d rats was significantly lower than that of the control rats and the significance lasted for about three weeks (Figure 1E). Furthermore, the cumulative contact time of M-1d and M-8d rats was significantly shorter than that of the control group (Figure 1F). However, at the fourth week after 7 consecutive days of IS stimulation, the total contact time of M-29d rats returned to the level of control rats (Figure 1E). Meanwhile, the total contact numbers were not found to be significantly different among the control, AH, M-1d, and M-8d rats (Figure 1G). This indicates that the significant reduction of total contact time in chronic migraine rats is not due to the decreases in animals’ attempts to drink milk but related to the periorbital hyperalgesia.

Subsequently, we applied traditional method, von Frey monofilaments, to confirm the change of periorbital threshold as previously described.^1^^,^^22^ The results of von Frey threshold of different headache rats were similar to those in orofacial stimulation test system. The von Frey threshold was not significantly different between control and AH rats (Figure 1H). After 7 consecutive days of IS stimulation, the von Frey threshold of M-1d and M-8d rats was significantly lower than that of control rats (Figure 1H) and recovered to the level of control rats on the 29th day (Figure 1H). These results suggest that the periorbital region of migraine rats generate hyperalgesia which lasted for a long time.

### The migraine-related anxiety in migraine model rats

**Figure 2:**
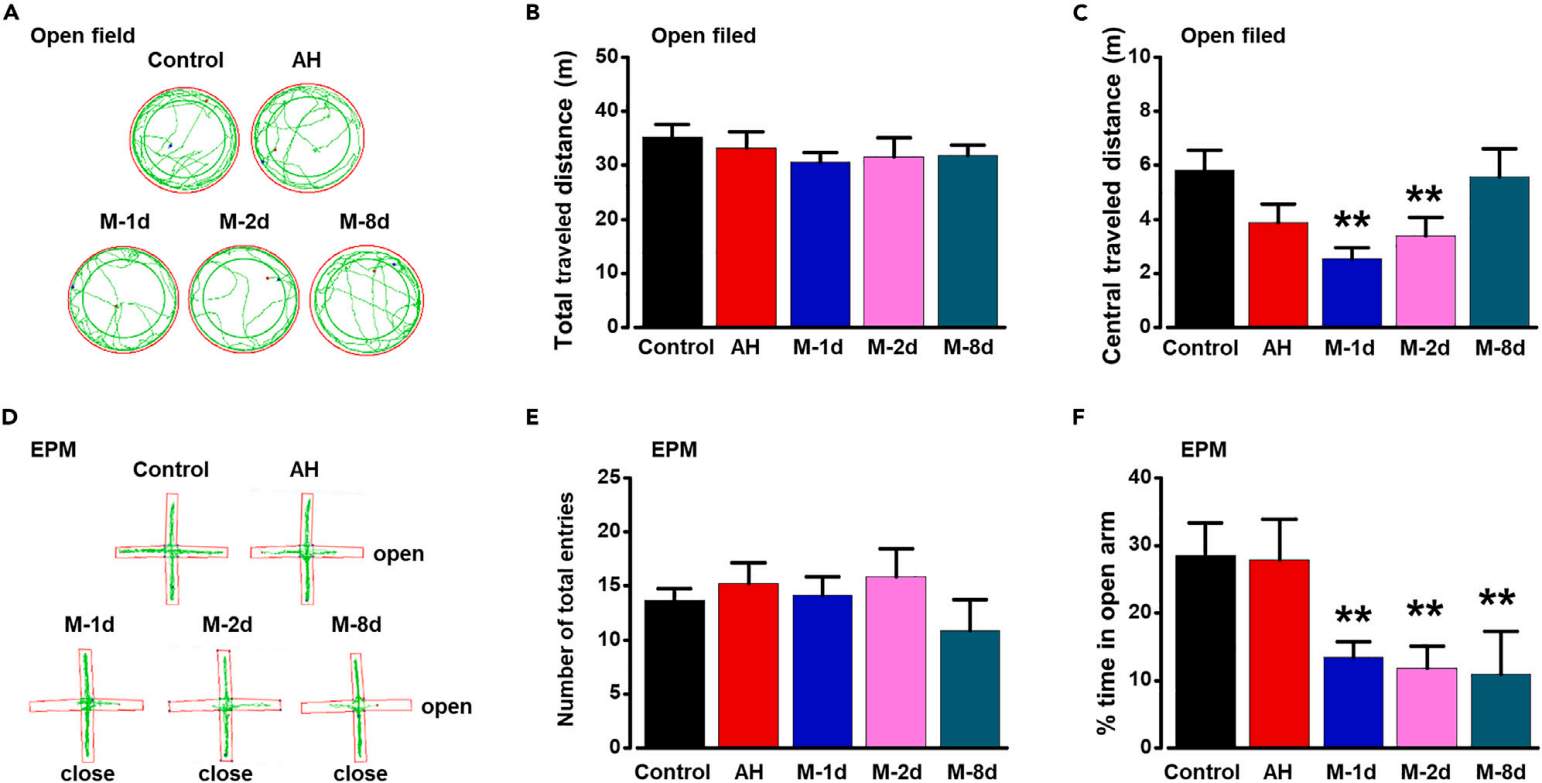
Anxiety-like behavior in different migraine model rats (A) Five samples of open field test from different rats. (B and C) Migraine rats showed anxiety-like behavior in open field test (Control: n = 8; AH: n = 8; M-1d: n = 8; M-2d: n = 8; M-8d rats: n = 7; *F*_(4, 34)_ = 3.867, p = 0.011, one-way ANOVA) but no difference in total traveled distance (*F*_(4, 34)_ = 0.480, p = 0.750, one-way ANOVA). (D) Five samples of EPM test from different rats. (E and F) Migraine rats showed anxiety-like behavior in EPM test (Control: n = 8; AH: n = 8; M-1d: n = 8; M-2d: n = 8; M-8d rats: n = 6; *F*_(4, 33)_ = 3.633, p = 0.015, one-way ANOVA) but no difference in total entries (*F*_(4, 33)_ = 0.772, p = 0.551, one-way ANOVA). *∗∗*p < 0.01 vs. control group. Data are represented as mean ± SEM.

Chronic pain has been reported to contribute to the generation of anxiety and other pain-related negative emotions.^15^^,^^23^ To test anxiety-related behavior in these groups, we performed open field and elevated plus maze (EPM) on these rats. We found that the anxiety behaviors occurred in chronic migraine rats. In the open field test, there was no difference in total traveled distance among control, AH, M-1d, M-2d, and M-8d rats (Figure 2B), but chronic migraine rats showed significantly less central traveled distance compared with that in control rats (Figure 2C). In EPM test, there was no difference in number of total entries among control, AH, M-1d, M-2d, and M-8d rats (Figure 2E), but migraine rats showed significantly less time in open arm compared with that in control rats (Figure 2F). In addition, acute headache did not affect the anxiety-related behavior of rats. These results suggest that long-term migraine attacks induce the anxiety-related behavior of rats, rather than a single acute headache.

### Phosphorylation levels of GluA1 and GluN2B enhanced in ACC in migraine

**Figure 3:**
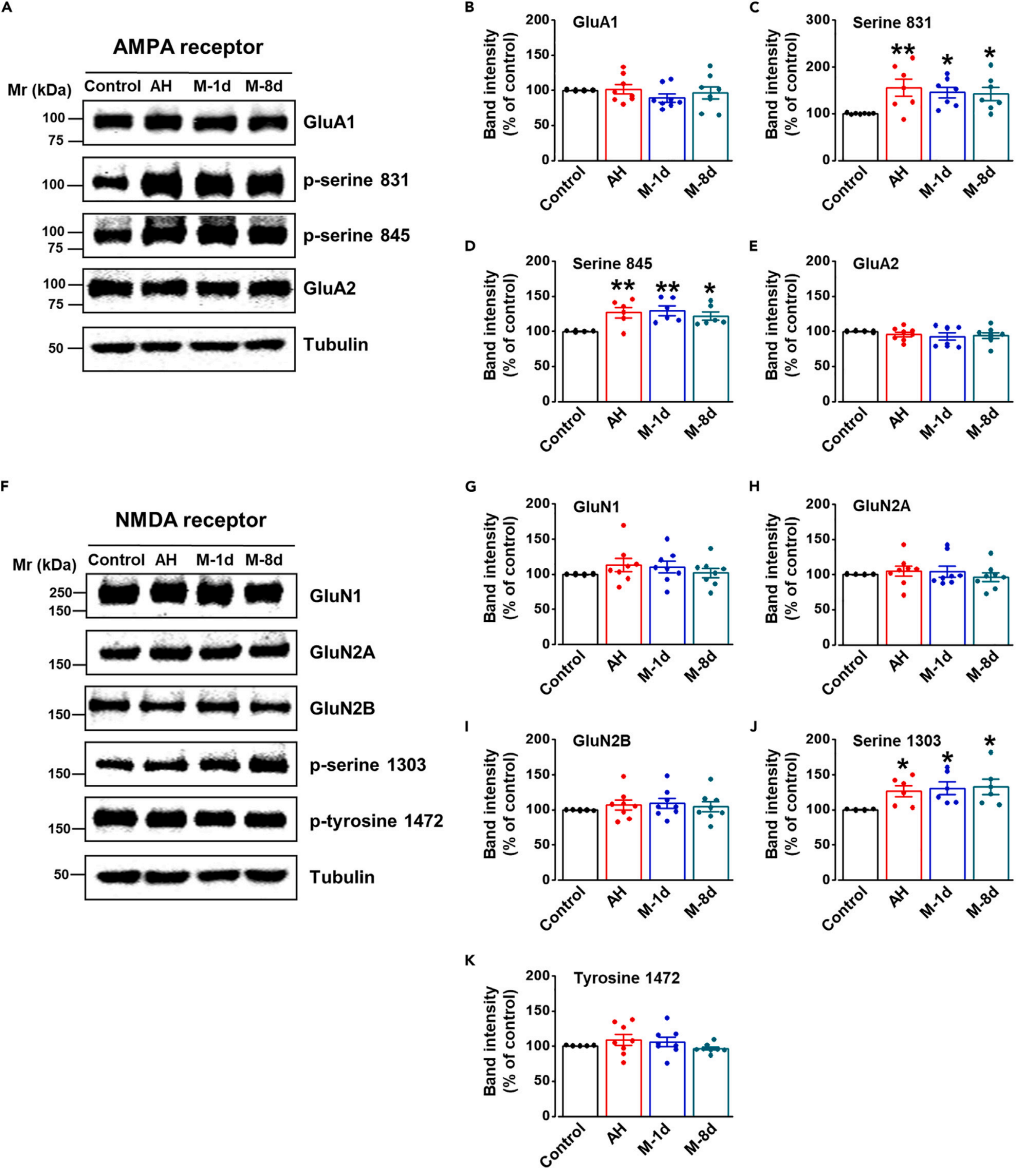
Enhanced phosphorylation levels of GluN2B and GluA1 occurred in ACC in migraine (A) Representative western blot for AMPA receptors in the ACC from control, AH, M-1d, and M-8d rats. (B–E) The total protein levels of GluA1 (n = 8 per group, *F*_(3, 28)_ = 0.781, p = 0.515, one-way ANOVA) and GluA2 (n = 8 per group, *F*_(3, 28)_ = 0.735, p = 0.540, one-way ANOVA) did not show difference among these groups, but the phosphorylation levels of GluA1 serine 831 (n = 7 per group, *F*_(3, 24)_ = 3.748, p = 0.024, one-way ANOVA) and serine 845 (n = 6 per group, *F*_(3, 20)_ = 5.404, p = 0.007, one-way ANOVA) significantly enhanced in AH, M-1d, and M-8d rats. (F) Representative western blot for NMDA receptors in the ACC from control, acute headache, M-1d, and M-8d rats. (G–K) The levels of GluN1 (n = 8 per group, *F*_(3, 28)_ = 0.847, p = 0.480, one-way ANOVA), GluN2A (n = 8 per group, *F*_(3, 28)_ = 0.412, p = 0.746, one-way ANOVA), GluN2B (n = 8 per group, *F*_(3, 28)_ = 0.411, p = 0.746, one-way ANOVA), and *p*-GluN2B-tyrosine 1472 (n = 8 per group, *F*_(3, 28)_ = 1.115, p = 0.360, one-way ANOVA) did not show difference among these groups, but the phosphorylation level of GluN2B serine 1303 significantly enhanced in AH, M-1d, and M-8d rats (n = 6 per group, *F*_(3, 20)_ = 3.400, p = 0.038, one-way ANOVA). *∗*p < 0.05, *∗∗*p < 0.01 vs. control group. Each data point represents an individual animal. Data are represented as mean ± SEM.

Both the AMPA receptor and NMDA receptor are tetramers composed of four subunits. Among them, GluN2B and GluA1 play a key role that contributes to the sensitization of the ACC in chronic pain.^8^ To determine whether these two receptors are involved in the ACC sensitization in migraine, we measured the levels of these two receptors in the ACC in migraine rats. We found the total protein levels of GluA1 (Figures 3A and 3B) and GluA2 (Figure 3E) didn’t show difference among control, AH, M-1d and M-8d rats. However, we found that the levels of phosphorylation of GluA1 serine 831 (Figures 3A and 3C) and serine 845 (Figure 3D) significantly increased in AH, M-1d, and M-8d rats compared with that in control rats.

Subsequently, we also tested the changes of NMDA receptors. We found that the total protein levels of GluN1 (Figure 3G), GluN2A (Figure 3H), and GluN2B (Figure 3I) did not change among control, AH, M-1d, and M-8d rats. However, we found that the level of GluN2B serine 1303 phosphorylation (Figures 3F and 3J) significantly increased in AH, M-1d, and M-8d rats, but not GluN2B tyrosine 1472 (Figure 3K). These results suggest that the NMDA receptor and AMPA receptor contribute to the ACC sensitization mainly through the phosphorylation sites of GluN2B and GluA1.

### Both long-term presynaptic and postsynaptic amplifications of migraine rats in the ACC

**Figure 4:**
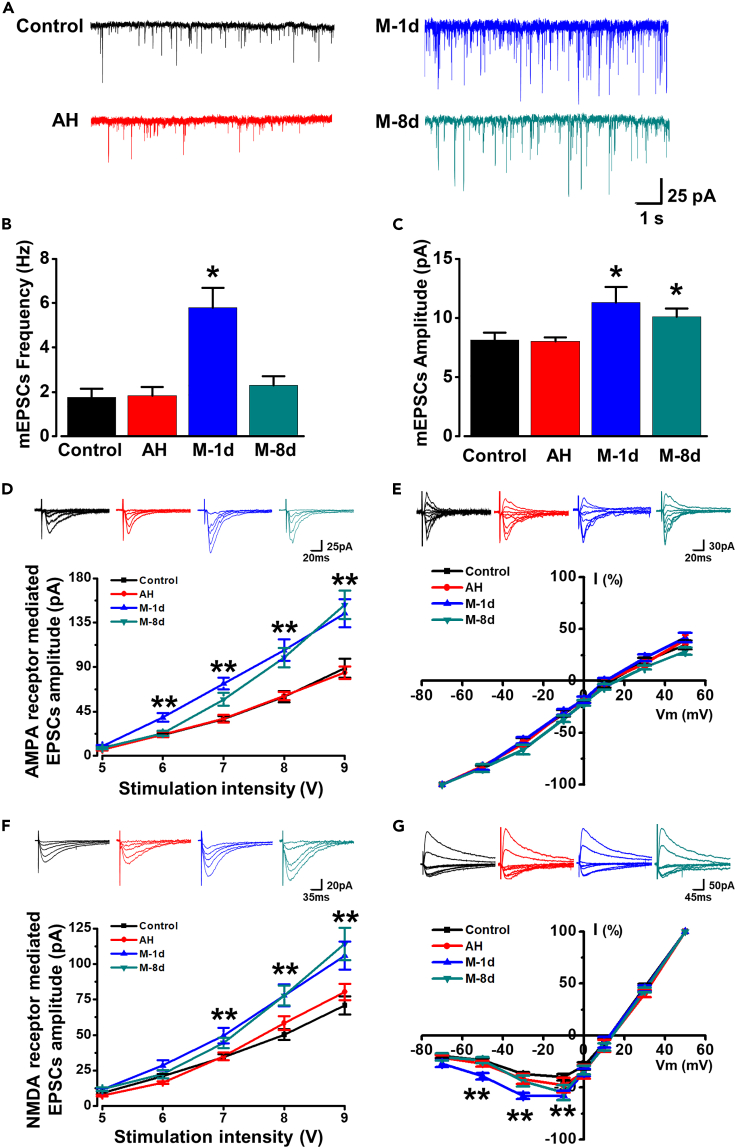
Characterization of whole-cell patch in the ACC of different rats (A–C) Statistical results of frequency (AH: *t*_(30)_ = −0.138, p = 0.891, unpaired *t* test; M-1d: *t*_(32)_ = −2.078, p = 0.046, unpaired *t* test; M-8d: *t*_(43)_ = −0.916, p = 0.365, unpaired *t* test) and amplitude (AH: *t*_(30)_ = 0.149, p = 0.882, unpaired *t* test; M-1d: *t*_(32)_ = −2.195, p = 0.036, unpaired *t* test; M-8d rats: *t*_(43)_ = −2.027, p = 0.049, unpaired *t* test) of the mEPSCs (n = 17 neurons/8 control rats, 15 neurons/5 AH rats, 17 neurons/6 M-1d rats, and 28 neurons/8 M-8d rats). (D) Pooled data showed the input-output curves of AMPA receptor-mediated EPSCs were shifted to the left in M-1d (n = 15 neurons/6 rats, *F*_(1, 145)_ = 49.962, p = 6.105E-11, two-way ANOVA) and M-8d rats (n = 15 neurons/6 rats, *F*_(1, 145)_ = 31.257, p = 1.089E-07, two-way ANOVA) compared with that in control rats (n = 16 neurons/5 rats). Meanwhile, there is no difference between AH (n = 15 neurons/5 rats, *F*_(1, 145)_ = 0.136, p = 0.713, two-way ANOVA) and control rats. (E) AMPA receptor-mediated I-V curves were not different in the ACC neurons among these groups (n = 20 neurons/8 control rats, 15 neurons/5 AH rats, 15 neurons/7 M-1d rats, and 17 neurons/7 M-8d rats, *F*_(3, 464)_ = 1.468, p = 0.223, two-way ANOVA). (F) Input-output curves of NMDA receptor-mediated EPSCs were shifted to the left in M-1d (n = 14 neurons/8 rats, *F* _(1, 145)_ = 31.594, p = 9.441E-08, two-way ANOVA) and M-8d rats (n = 20 neurons/7 rats, *F*_(1, 175)_ = 24.718, p = 1.574E-06, two-way ANOVA) compared with that in control (n = 17 neurons/5 rats) and AH rats (n = 16 neurons/5 rats). (G) The I-V curves of NMDA receptor from acute headache, M-1d, and M-8d rats were also shifted to the left compared with that from control rats (n = 15 neurons/7 control rats, 16 neurons/5 AH rats, 13 neurons/7 M-1d rats, and 17 neurons/7 M-8d rats, *F*_(3, 456)_ = 6.492, p = 2.615E-04, two-way ANOVA). *∗*p *<* 0.05; *∗∗*p < 0.01 vs. control group. Data are represented as mean ± SEM.

To determine whether the synaptic transmission of ACC is enhanced in migraine rats, we recorded miniature excitatory postsynaptic currents (mEPSCs) by whole-cell patch-clamp method. Glutamate is the major fast excitatory transmitter in the ACC.^24^ The frequency of mEPSCs is most likely dependent entirely upon the probability of release from presynaptic elements.^25^^,^^26^^,^^27^ In the experiment of whole-cell patch-clamp electrophysiology (Figure 4), we found that the frequency of mEPSCs in M-1d group was significantly increased compared with that in control group (Figure 4B). Therefore, we infer that the presynaptic release of glutamate was enhanced after the sustained inflammation stimulation of dura. However, this enhancement did not last too long, because we found that there was no difference in the frequency of mEPSCs between control and M-8d rats (Figure 4B). In addition, both of the amplitudes in M-1d and M-8d rats were significantly larger than that in control rats (Figure 4C). Meanwhile, neither the frequency nor the amplitude of the mEPSCs changed between control and acute headache rats (Figures 4A–4C). These results suggest that presynaptic and postsynaptic factors are involved in the enhancement of ACC transmission in migraine rats, even if the time of their involvement is not synchronized.

Postsynaptic AMPA and NMDA receptor-mediated currents play crucial roles in the induction and maintenance of ACC long-term potentiation (LTP) in chronic pain.^8^ To determine which receptor is associated with the enhanced transmission in ACC in migraine rats, we recorded the input (stimulation intensity)-output (EPSC amplitude) efficiency and I-V relationship of the AMPA and NMDA receptor-mediated synaptic responses. Compared with control rats, the AMPA receptor-mediated input-output (I-O) curve in M-1d rats was shifted to the left (Figure 4D) and this result was similar to that in M-8d rats (Figure 4D), while there was no difference in the I-O curves between control and AH rats (Figure 4D). These results indicate that the AMPA receptor-mediated excitatory responses are potentiated in migraine rats. However, there was no significant difference in the I-V curves (−70 to +50 mV) among these groups (Figure 4E).

We then tested NMDA receptor-mediated currents in these rats. The results of I-O curves of NMDA receptor were similar to that of AMPA receptor. We found that the NMDA receptor-mediated I-O curves in M-1d and M-8d rats were shifted to the left compared with that in control rats (Figure 4F). Furthermore, the I-V curves of NMDA receptor from AH, M-1d, and M-8d rats were also shifted to the left compared with that from control rats (Figure 4G). These results suggest that both of NMDA and AMPA receptor-mediated synaptic transmission are enhanced in migraine model rats.

### LTP was failed to be induced in the ACC of migraine rats

**Figure 5:**
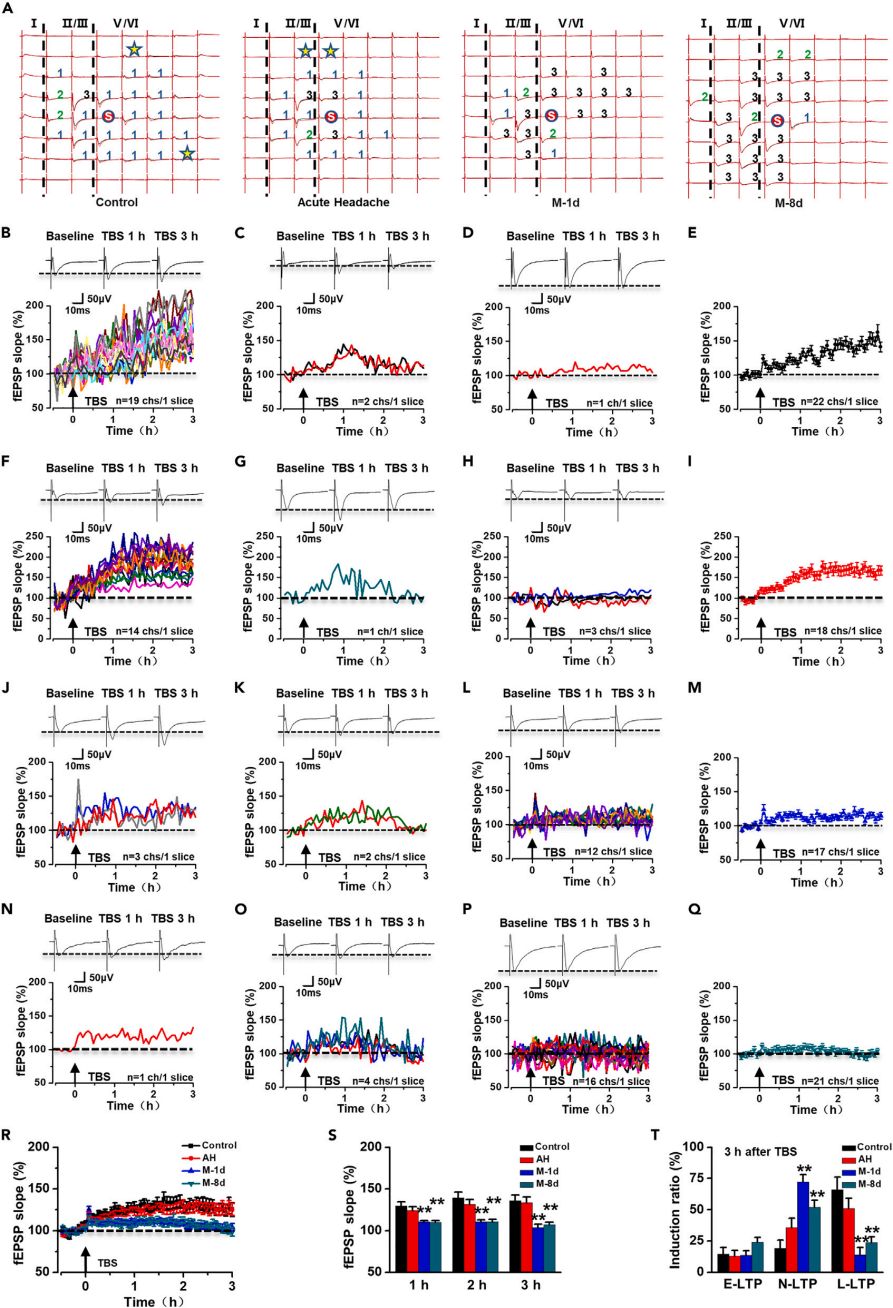
Repeated daily IS stimulation occluded TBS-induced LTP in ACC (A) Four samples showed the network fEPSP in the ACC of control, acute headache, M-1d, and M-8d rats. The fEPSPs were induced by electrical stimulation on one channel (marked as “s”) and were recorded from the other 63 channels 30 min before (black) and 3 h after (red) TBS. Asterisks indicated the channels with recruited fEPSPs in the ACC. Superimposed sample traces at different time points (30 min before and 3 h after TBS) showed the following three types of plasticity: channels showing L-LTP (1), channels showing E-LTP (2), and channels without potentiation (3). (B–D) The temporal changes of fEPSP slopes from one sample slice from a control rat: 19 channels with L-LTP (B), 2 channels with E-LTP (C), and 1 channel without potentiation (D). (E) The final averaged slopes for all 22 activated channels at 3 h after TBS in the control rat (148.95 ± 7.89% of the baseline). (F–H) The temporal changes of fEPSP slopes from one sample slice from an AH rat: 14 channels with L-LTP (F), 1 channel with E-LTP (G), and 3 channels without potentiation (H). (I) The final averaged slopes for all 18 activated channels at 3 h after TBS in the AH rat (165.86 ± 11.56% of the baseline). (J–L) The temporal changes of fEPSP slopes from one sample slice from an M-1d rat: 3 channels with L-LTP (J), 2 channels with E-LTP (K), and 12 channels without potentiation (L). (M) The final averaged slopes for all 17 activated channels at 3 h after TBS in the M-1d rat (113.85 ± 4.59% of the baseline). (N–P) The temporal changes of fEPSP slopes from one sample slice from an M-8d rat: 1 channel with L-LTP (B), 4 channels with E-LTP (C), and 16 channels without potentiation (D). (Q) The final averaged slopes for all 21 activated channels at 3 h after TBS in the M-8d rat (99.12 ± 5.03% of the baseline). (R) The averaged fEPSP slopes of all recorded channels in control (11 slices/5 rats), AH (15 slices/7 rats), M-1d (16 slices/6 rats), and M-8d (28 slices/9 rats) rats. (S) The averaged fEPSP slopes from total active channels of control, AH (1 h: *t*_(24)_ = 0.869, p = 0.393, unpaired *t* test; 2 h: *t*_(24)_ = 0.872, p = 0.392, unpaired *t* test; 3 h: *t*_(24)_ = 0.231, p = 0.819, unpaired *t* test), M-1d (1 h: *t*_(25)_ = 4.548, p = 1.201E-04, unpaired *t* test; 2 h: *t*_(25)_ = 4.685, p = 8.429E-05, unpaired *t* test; 3 h: *t*_(25)_ = 4.428, p = 1.641E-04, unpaired *t* test), and M-8d (1 h: *t*_(37)_ = 4.550, p = 5.602E-05, unpaired *t* test; 2 h: *t*_(37)_ = 4.699, p = 3.558E-05, unpaired *t* test; 3 h: *t*_(37)_ = 4.395, p = 8.967E-05, unpaired *t* test) rats at different time points after the TBS. (T) M-1d and M-8d rats showed fewer L-LTP channels (M-1d: *t*_(25)_ = 4.796, p = 6.327E-05, unpaired *t* test; M-8d: *t*_(37)_ = 4.424, p = 8.222E-05, unpaired *t* test) and more N-LTP channels (M-1d: *t*_(25)_ = −6.000, p = 2.885E-06, unpaired *t* test; M-8d: *t*_(37)_ = −3.419, p = 0.002, unpaired *t* test) at 3 h after TBS compared with those in control and AH rats. *∗∗*p < 0.01 vs. control group. Data are represented as mean ± SEM.

LTP is a key synaptic mechanism for chronic pain.^8^ To determine whether acute headache is sufficient to induce LTP in ACC, we applied the MED 64 system to record the network LTP in the control and AH rats. 19 activated channels showed late phase of LTP (L-LTP) which lasted for 3 h after LTP induction in the sample slice of control rats, while only 2 activated channels showed early phase of LTP (E-LTP) lasting less than 3 h and 1 activated channel showed none LTP (N-LTP). The final averaged slope of all 22 activated channels was 148.95 ± 7.89% of the baseline at 3 h after LTP induction (Figures 5B–5E). The results of AH rats were similar to that of control rats. In a typical sample slice of AH rats, there were 14 activated channels that showed L-LTP, 1 activated channel showed E-LTP, and 3 activated channels showed N-LTP. The final averaged slope of all 18 activated channels was 165.86 ± 11.56% of the baseline at 3 h after LTP induction (Figures 5F–5I).

After analyzing all 144 activated channels from 11 slices of 5 control rats, we found that the averaged induction rate of three different types of responses was 66.07 ± 10.19% (L-LTP), 14.75 ± 5.15% (E-LTP), and 19.18 ± 6.37% (N-LTP), respectively (Figure 5T). The final averaged slope of all 144 activated channels was 135.80 ± 6.99% of the baseline at 3 h (Figures 5R and 5S). From 15 slices of 7 AH rats, 51.21 ± 7.88% channels showed L-LTP (Figures 5T), 12.94 ± 4.68% channels showed E-LTP (Figures 5T), and 35.84 ± 7.43% channels failed to potentiation (Figure 5T). At 3 h after LTP induction, the final averaged slope of all 204 activated channels in AH rats was also similar to that in control rats (Figures 5R and 5S). These results indicate that acute headache is not sufficient to induce LTP in ACC.

To investigate whether synaptic plasticity in the ACC of migraine rats has changed, we also recorded network LTP in M-1d rats. Figures 5J–5M showed a typical sample slice with 17 activated channels of the M-1d rats. There were only 3 activated channels that showed L-LTP. Meanwhile, 2 activated channels showed E-LTP and 12 activated channels showed N-LTP. The final averaged slope of the all 17 activated channels was 113.85 ± 4.59% of the baseline at 3 h after LTP induction (Figure 5M). The results from 16 slices of 6 M-1d rats showed that the averaged induction rate of different types of channels in M-1d rats was also affected. M-1d rats showed more channels with N-LTP (72.25 ± 5.88%; Figure 5T) and fewer channels with L-LTP (14.16 ± 5.65%; Figure 5T). The final averaged slope of all 167 activated channels in M-1d rats was significantly lower than that in control rats (M-1d rats, 103.86 ± 3.60% of the baseline; Figures 5R and 5S). All of these data suggest that potentiation of synaptic connections happens in ACC of migraine rats.

### Migraine caused long-term occlusion on the TBS-induced LTP in the ACC

To determine whether LTP can be maintained in ACC of migraine rats for a long time without IS stimulation, we recorded the network LTP in M-8d rats. As shown in Figures 5N–5Q, the numbers of different types of channels in a typical slice with 21 activated channels from M-8d rats were similar to those in M-1d rats. After LTP induction, there was 1 L-LTP channel, 4 channels with E-LTP, and 16 channels that showed no potentiation, in this sample slice. Meanwhile, the final averaged slope of this typical sample from M-8d rats was 99.12 ± 5.03% of the baseline at 3 h after LTP induction (Figure 5Q). Our results from a total of 347 activated channels from 28 slices of 9 M-8d rats showed that the induction rate of L-LTP channel in M-8d rats was significantly lower than that in the control rats (M-8d rats, 23.86 ± 4.48%; Figure 5T), while the induction rate of N-LTP channel in M-8d rats was significantly higher than that in the control rats (M-8d rats, 51.99 ± 5.45%; Figure 5T). Furthermore, the final averaged slope of all 347 activated channels in M-8d rats was also significantly lower than that in control rats (M-8d rats, 107.01 ± 3.08% of the baseline; Figures 5R and 5S). These results suggest that migraine causes long-term occlusion on the theta-burst stimulation (TBS)-induced LTP in the ACC without IS stimulation and such long-term occlusion might contribute to the long-term hyperalgesia in migraine rats.

### Recruited responses in the ACC of control and acute headache rats but not in migraine rats

**Figure 6:**
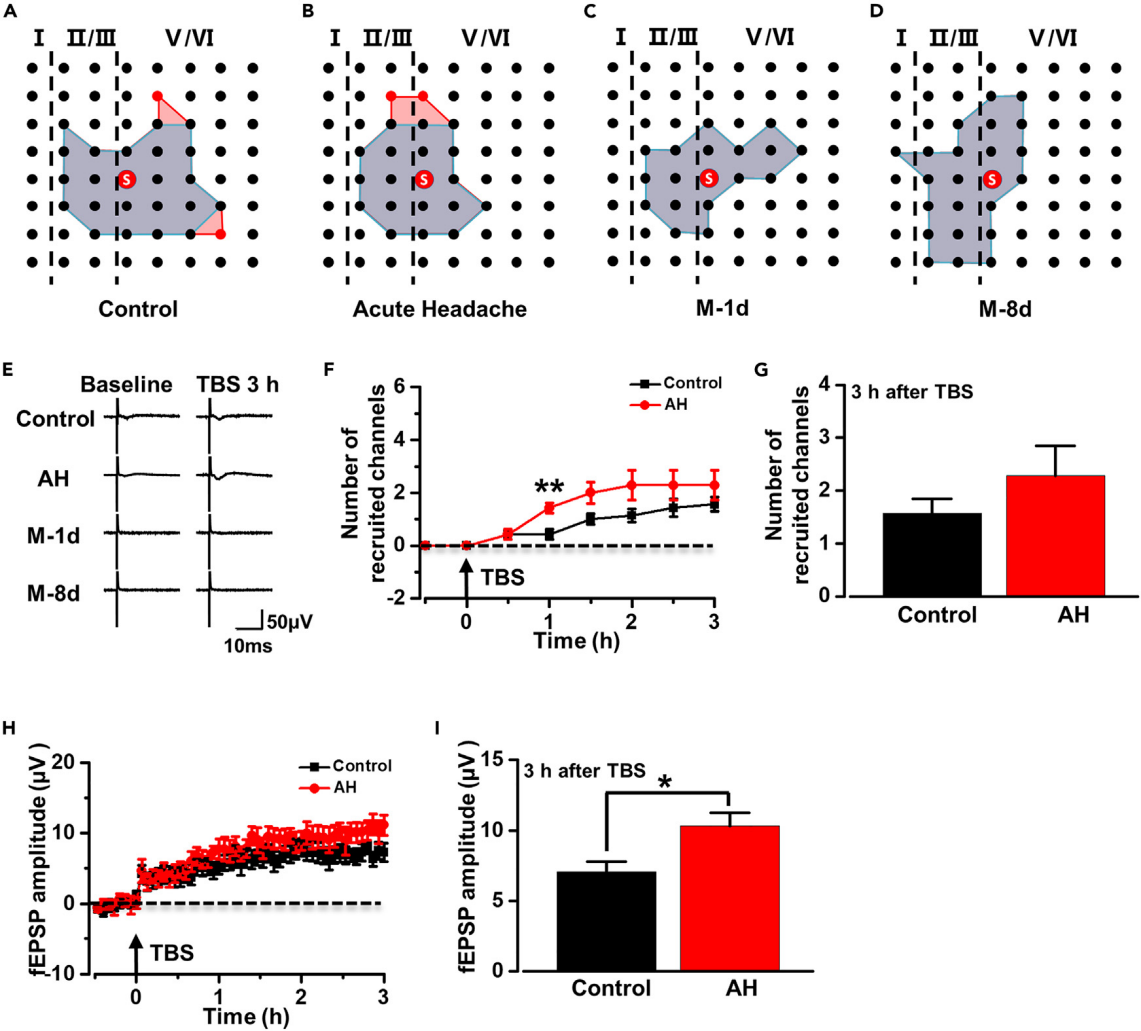
Recruited responses induced by TBS in the ACC of control and AH rats (A–D) Sample slices showed the distribution of the basal activated channels (blue) and the TBS-recruited channels (red) in control rats (A) and AH rats (B) but not in M-1d rats (C) or M-8d rats (D). (E) Superimposed samples showed the recruited responses induced by TBS. (F and G) The temporal changes of the number of the recruited fEPSPs in control and AH rats (n = 7 slices/4 rats/group, *t*_(12)_ = −3.500, p = 0.004, unpaired *t* test). (H and I) The temporal changes of the amplitude of recruited channels in control and AH rats (n = 7 slices/4 rats/group, *t*_(12)_ = −2.570, p = 0.025, unpaired *t* test). *∗*p < 0.05; *∗∗*p < 0.01 vs. control group. Data are represented as mean ± SEM.

Previous studies have shown that silent synapses can be recruited by postsynaptic trafficking of the AMPA receptor, and such recruitment can be observed by a multichannel recording system.^28^^,^^29^ To investigate the effect of different types of headaches on recruited responses, we analyzed the number and the amplitude of recruited channels from rats with different types of headaches. 12.82 ± 2.14 activated channels per slice were observed in control rats during the baseline recording (Figure 6A). Although recruited responses could not be recorded in all slices, 1.57 ± 0.30 recruited channels were recorded at 3 h after TBS in the slices of control rats that generated recruited responses (Figures 6F and 6G), and the recruited channels were located on the edge of the basal active area (Figures 6A and 6B).

Similar to the result of control rats, there was no difference in the number of basal activated channels between control and AH rats; 2.29 ± 0.61 recruited channels per slice were observed in AH rats at 3 h after TBS (Figures 6F and 6G). However, there was an interesting phenomenon - more recruited responses were recorded in AH rats at 1 h after TBS compared with that in control rats (Figure 6F). As for chronic migraine rats, no recruited response was observed in M-1d and M-8d rats (Figures 6C and 6D).

Figure 6H shows the time course of the changed fEPSP amplitude of recruited channels. In control rats, the amplitude of recruited channels gradually increased after TBS and finally became 7.07 ± 0.77 μV at 3 h. However, the amplitude of AH rats at 3 h after TBS was 10.34 ± 1.01 μV which was larger than that in the control rats (Figures 6H and 6I). These results indicate that headache stimulation induced postsynaptic trafficking of AMPA receptor in silent synapses, and such trafficking might happen within 1 h after headache stimulation.

### Effect of AC1 on mechanical withdrawal hyperalgesia in migraine rats

**Figure 7:**
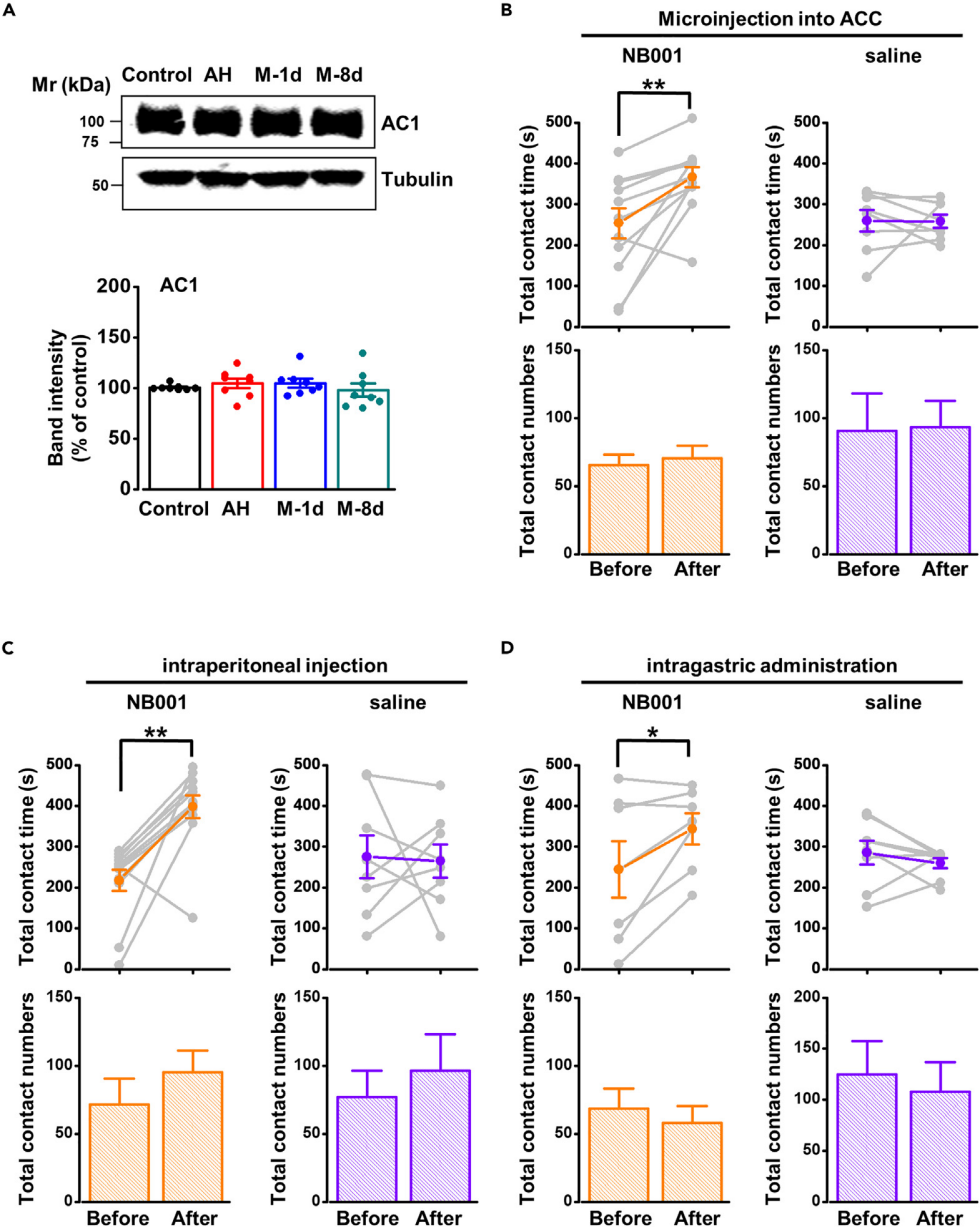
Effect of AC1 on periorbital hyperalgesia in migraine rats (A) The total protein level of AC1 did not change among control, AH, and migraine rats (n = 8 per group, *F*_(3, 28)_ = 0.512, p = 0.677, one-way ANOVA). (B–D) Inhibition of AC1 effectively relieved periorbital hyperalgesia in M-8d rats by microinjection into ACC (n = 12 per group, *t*_(11)_ = −3.526, p = 0.005, paired *t* test), intraperitoneal injection (n = 12 per group, *t*_(11)_ = −4.682, p = 0.001, paired *t* test), or intragastric administration (n = 7 per group, *t*_(6)_ = −2.558, p = 0.043, paired *t* test), but did not affect the contact times (microinjection into ACC: *t*_(11)_ = −2.088, p = 0.061, paired *t* test; intraperitoneal injection: *t*_(11)_ = −1.382, p = 0.194, paired *t* test; intragastric administration: *t*_(6)_ = 0.834, p = 0.436, paired *t* test). *∗*p < 0.05, *∗∗*p < 0.01 vs. control group. Data are represented as mean ± SEM.

Recent studies indicate that the total protein level of AC1 upregulates in visceral pain and the upregulation of AC1 is important for chronic pain.^8^^,^^19^^,^^30^ Therefore, we tested the level of AC1 in the ACC in migraine to see the changes of AC1 in chronic migraine rats. We found that the total protein level of AC1 did not show a difference among control, AH, M-1d, and M-8d rats (Figure 7A). This result indicates that migraine does not affect total protein level of AC1, which is different from neuropathic pain or visceral pain.

We then applied the AC1 antagonist, NB001, on M-8d rats to determine whether AC1 is involved in migraine in an activity-dependent form. We found that the microinjection of NB001 into the ACC of migraine rats effectively increased the duration of time drinking milk (Figure 7B upper left), but did not affect total contact numbers (Figure 7B bottom left). We also applied intraperitoneal injections and intragastric administration of NB001 on migraine rats. We found that applications of NB001 at both 20 (i.p., Figure 7C upper left) and 60 mg/kg (i.g., Figure 7D upper left) also effectively improved the duration of milk drinking in migraine rats, but did not affect total contact numbers (Figure 7C bottom left and Figure 7D bottom left). However, low-dose NB001 (i.p., 5 mg/kg) did not improve the periorbital hyperalgesia of migraine rats (Figure S1B).

### Effect of AC1 on migraine-related anxiety

**Figure 8:**
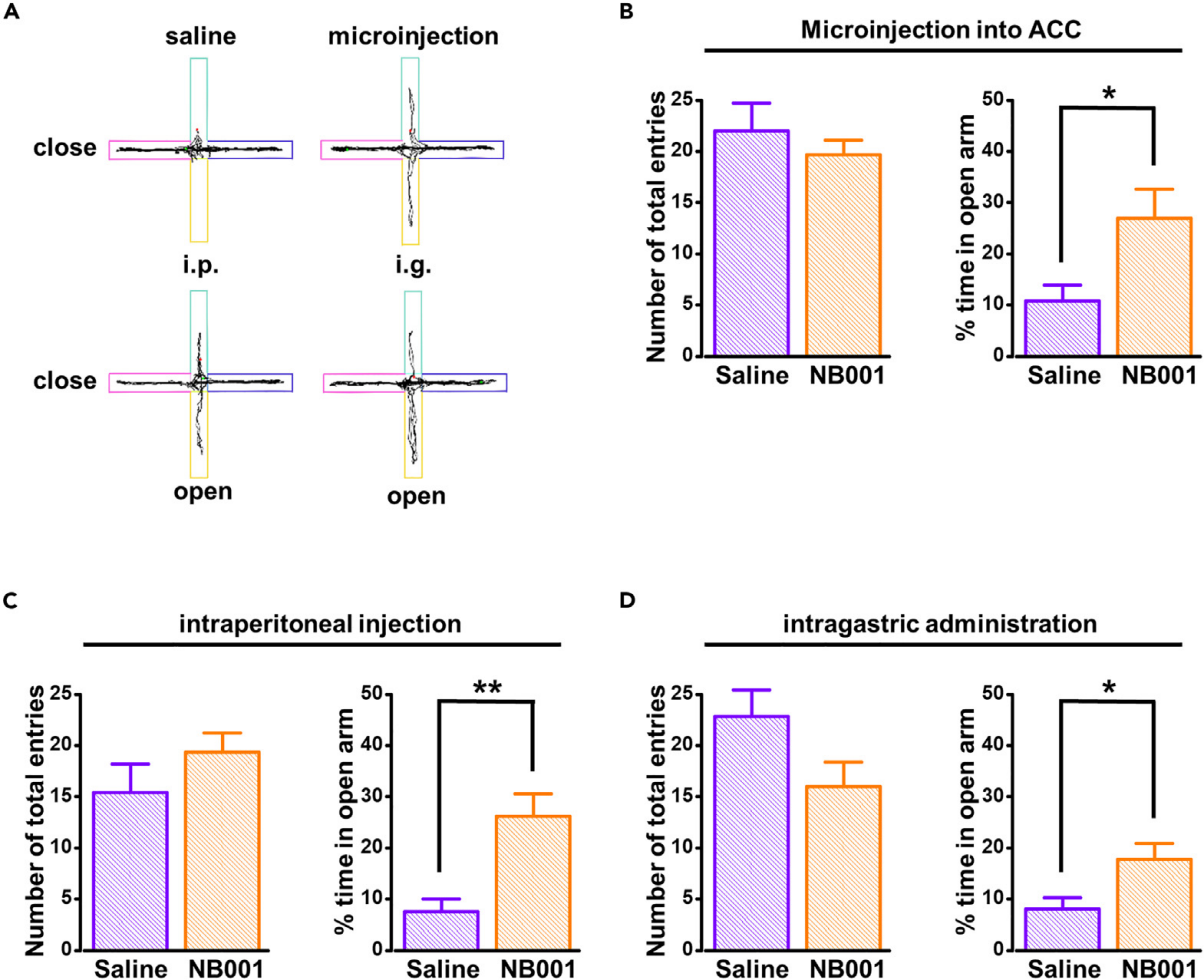
Effect of AC1 on migraine-related anxiety behavior in migraine rats (A) Four samples of EPM test from M-8d rats that received saline or NB001 injection. (B–D) Inhibition of AC1 effectively relieves migraine-related anxiety behavior in M-8d rats by microinjection into ACC (n = 6 per group, *t*_(10)_ = −2.543, p = 0.029, unpaired *t* test), intraperitoneal injection (M-8d + saline: n = 7; M-8d + NB001: n = 6; *t*_(11)_ = −3.858, p = 0.003, unpaired *t* test), or intragastric administration (n = 6 per group, *t*_(10)_ = −2.529, p = 0.030, unpaired *t* test), but did not affect the total enter numbers (microinjection into ACC: *t*_(10)_ = 0.765, p = 0.462, unpaired *t* test; intraperitoneal injection: *t*_(11)_ = −1.132, p = 0.282, unpaired *t* test; intragastric administration: *t*_(10)_ = 1.938, p = 0.081, unpaired *t* test). *∗*p < 0.05, *∗∗*p < 0.01 vs. control group. Data are represented as mean ± SEM.

Finally, we also tested the effect of AC1 on migraine-related anxiety behaviors. Microinjection NB001 into ACC significantly increased the time of M-8d rats in open arm (Figure 8B right), but did not affect the total enter numbers (Figure 8B left). Intraperitoneal injection and intragastric administration also showed similar results with microinjection (Figures 8C and 8D). These findings suggest that AC1 is involved in migraine and migraine-related anxiety through its own activity changes, rather than via protein synthesis. This is different from previous studies about neuropathic pain or visceral pain.^8^^,^^30^^,^^31^

## Discussion

**Figure 9:**
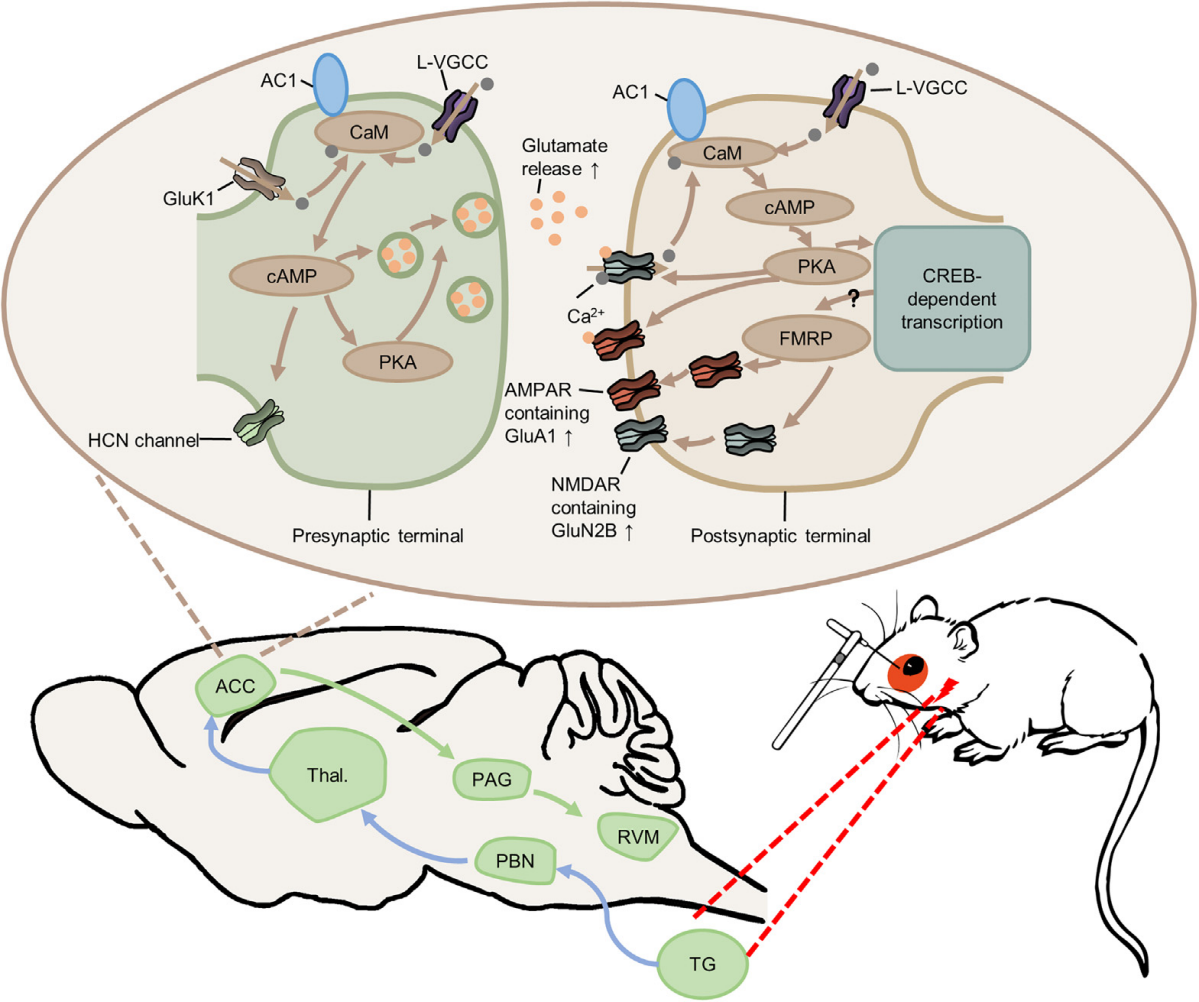
A synaptic model for migraine and migraine-related anxiety in ACC Sensory inputs from periphery (such as periorbital region) active TG neurons and further project (blue arrows) to cortices (including ACC and IC) through PBN and thalamus, resulting in increased excitability in the ACC. The increased excitability in the ACC includes the increase of presynaptic release of glutamate, and enhanced postsynaptic responses of AMPA and NMDA receptors. In addition, FMRP, which indicate previously in visceral pain, may also contribute to the enhanced responses of AMPA and NMDA receptors. Subsequently, presynaptic and postsynaptic plasticity changes in ACC further contribute anxiety and cutaneous sensitization in chronic migraine rats through descending sensory pathway (green arrows). ACC, anterior cingulate cortex; AC1, adenylyl cyclase 1; AMPAR, AMPA receptor; CaM, calmodulin; cAMP, cyclic adenosine monophosphate; CREB, cAMP response element-binding protein; FMRP, fragile X mental retardation protein; GluK1, kainate receptor; HCN, hyperpolarization-activated cyclic nucleotide-gated; L-VGCC, L-type voltage-gated calcium channel; NMDAR, NMDA receptor; PAG, periaqueductal gray; PBN, parabrachial nucleus; PKA, cyclic AMP-dependent protein kinase; RVM, rostroventral medulla; TG, trigeminal ganglion.

Recent studies using animal models of chronic pain have consistently demonstrated that ACC plasticity is important for behavioral sensitization and emotional anxiety,^8^ and inhibiting AC1 may serve as a novel approach for treating neuropathic pain, cancer pain, visceral pain, and inflammatory pain.^19^^,^^30^^,^^32^ In the present study, we demonstrate that AC1 plays a critical role in chronic migraines. Both phosphorylation of NMDA and AMPA receptors in the ACC have been found after the inflammation stimulation of dura, and presynaptic release of glutamate and postsynaptic responses of AMPA and NMDA receptors were enhanced. Our behavioral studies found that inhibition of ACC excitability by local administration of AC1 inhibitor NB001 significantly alleviated anxiety and inhibited hyperalgesia. To our knowledge, this is the first time that it has been demonstrated that AC1-dependent ACC plasticity plays a critical in chronic migraines (Figure 9).

### Behavioral hyperalgesia in chronic migraine

Cutaneous sensitization, or allodynia, is a typical symptom of the migraineur.^2^^,^^33^^,^^34^ Previous studies have demonstrated that there is a diffusible allodynia (including contralateral head and legs) in migraine rats.^1^^,^^5^ In the present study, we confirmed this through an improved orofacial stimulation test system. We found that the periorbital hyperalgesia lasted for about 3 weeks without persistent inflammatory stimulation. Previous studies using animal models of inflammatory pain or neuropathic pain have found that the postsynaptic potentiation (or called post-LTP) of ACC contributes to the peripheral sensitization of rodents with chronic pain.^8^^,^^18^ In this study, we found that excitatory postsynaptic transmission in ACC of chronic migraine rats was significantly enhanced. Postsynaptic AMPA receptor-mediated currents and the amplitude of mEPSCs were increased in the ACC. In keeping with these findings, we discovered that TBS-induced post-LTP in the ACC of migraine rats was occluded. Biochemical experiments showed that phosphorylation levels of AMPA GluA1 (including serine 831 and serine 845) were increased in the ACC of migraine rats. Our previous studies showed that increased phosphorylation of GluA1 is important for post-LTP in the ACC.^29^^,^^35^ Therefore, our results strongly suggest that the cortical mechanism of AMPA receptor regulation plays an important role in chronic migraine, as well as chronic pain triggered by somatosensory injuries.^8^^,^^18^

In addition to the enhancement of the AMPA receptor, we found that NMDA receptor-mediated postsynaptic currents were also enhanced in chronic migraine rats. Furthermore, phosphorylation of GluN2B was found to be increased. Recent studies have shown that the phosphorylation of NMDA GluN2B receptors contributes to cortical excitation and chronic pain of somatic and visceral pain.^36^^,^^37^^,^^38^ For example, the function of NMDA receptor in ACC has been reported to be enhanced in a mouse model of visceral pain, mainly through increased phosphorylation of GluN2B at serine 1303 and tyrosine 1472.^37^ In this study, we found increased phosphorylation of GluN2B only at the site of serine 1303, but not tyrosine 1472, in the ACC of chronic migraine rats. The difference may be due to different signaling pathways and different pain models. In addition, the selective increased phosphorylation of GluN2B tyrosine 1472 has been reported in trigeminal nucleus caudalis of the trigeminovascular model.^38^ These findings indicate that different sites of GluN2B phosphorylation in migraine are related to the various function of NMDA receptors in different central regions.

It is known that spinal pain transmission receives descending modulation from supraspinal structures including brainstem, periaqueductal gray, and cortex.^39^ In addition to cortical excitation, recent studies reported that the ACC also exerts a strong descending facilitation of spinal pain transmission.^39^^,^^40^^,^^41^ It is quite possible that ACC-spinal facilitation may contribute to behavioral allodynia in chronic migraine. ACC excitation reported in this study may affect spinal pain transmission through descending facilitation modulation.^40^ Previous studies about descending modulation in migraine have mainly focused on descending inhibition pathways from the lower brainstem level (such as rostral ventromedial medulla and PAG).^2^^,^^42^ Future studies are clearly needed to reveal the descending facilitation modulation from ACC for migraine (Figure 9).

### Emotional anxiety in chronic migraine

Previous clinical studies have demonstrated that patients with migraine suffer from anxiety, and anxiety could enhance migraine.^6^^,^^43^ However, the exact neuronal mechanism for the interaction between migraine and anxiety is unclear. In this study, we found that there is long-lasting anxiety in chronic migraine rats. Previous studies only measured anxiety behaviors immediately after inflammatory stimulation, but did not examine the long-lasting anxiety behaviors.^44^^,^^45^ Here, we found that the long-lasting anxiety behaviors can last for at least one week in chronic migraine rats without persistent inflammatory stimulation of dura. Since we detected presynaptic enhancement of glutamate release in chronic migraine, it is likely that this presynaptic enhancement contributes to behavioral anxiety. It has been previously demonstrated that AC1-dependent pre-LTP in ACC is important for injury-induced anxiety.^8^^,^^15^ Consisting with this finding, we found that that the microinjection of AC1 inhibitor into the ACC alleviated anxiety in migraine rats.

### Clinical implication

Both triptans and calcitonin gene-related peptide (CGRP) receptor antagonists—two commonly used peripheral drugs for the treatment/prevention of acute migraine—are reported to be less effective in cutaneous sensitization and anxiety in humans or rodents with chronic migraines.^46^^,^^47^^,^^48^^,^^49^ AC1, a neuronal form of subtype ACs, has been shown to contribute to both pre-LTP and post-LTP in the ACC.^8^^,^^50^^,^^51^^,^^52^ In this study, we found that NB001, applied locally in the ACC or orally, rapidly generated significant analgesic and anti-anxiety effect in chronic migraine rats, suggesting the AC1-dependent ACC plasticity contributes to migraine. This finding is consistent with our previous studies that AC1 is critical for the plasticity of ACC, and knockout or inhibition of AC1 can alleviate pain and anxiety behaviors in different animal models of chronic pain.^19^^,^^30^^,^^51^ Our recent studies reported that phosphorylation levels of AMPA GluA1 mediated by cAMP pathway is crucial to synaptic transmission in the ACC, and CGRP is also likely to act through cAMP pathway to enhance excitatory transmission in the ACC.^29^^,^^53^ Furthermore, human studies showed that increasing cAMP level by inhibiting phosphodiesterase in human can enhance the percentage of headache events.^54^^,^^55^^,^^56^ Thus, as we reported in this study, direct inhibition of AC1 or AC1 downstream may be more effective in the treatment of migraine. Recently, CGRP antagonists have been approved to treat spontaneous pain and allodynia for the prevention of migraine.^49^^,^^57^^,^^58^ However, they caused side effects such as liver toxicity.^59^^,^^60^ Differing from CGRP antagonists, our previous animal data as well as phase I clinical studies found that NB001 is very safe in both animals and humans.^19^^,^^30^^,^^61^ The superiority of NB001 in the treatment of migraine lies in that it did not interfere with normal synaptic transmission, but only inhibit the enhanced synaptic transmission under the condition of chronic pain. Hansen et al. detected the anxiety behavior (elevated plus maze), motor function (Rota-rod), and fear memory after the application of NB001 in healthy adult mice, and they found NB001 has no obvious side effect.^19^ In human studies, no serious adverse event was reported in healthy candidates after the oral administration of NB001.^61^ We believe that AC1 can serve as a new drug target for the treatment of chronic migraine, and NB001 is a strong drug candidate and could meet the need for better drugs.

### Limitations of the study

Although our study provides strong evidence that LTP in the ACC is involved in the onset and progression of migraine, as well as in migraine-related headache and anxiety, there are still some unanswered questions in this study that warrant further exploration. First of all, although we have demonstrated that LTP in the ACC is involved in the regulation of migraine and migraine-related anxiety, and this regulation is mediated by AC1, the upstream and downstream molecular signals of AC1 are still unclear and require further investigation. Furthermore, there are other forms of synaptic plasticity in ACC besides the postsynaptic NMDA receptor-dependent LTP in ACC that we report here, but we cannot rule out the involvement of other forms of synaptic plasticity in the ACC in the modulation of migraine, such as long-term depression within the ACC. Finally, as mentioned in the discussion, recent studies reported that the ACC also exerts a strong descending facilitation of spinal pain transmission. Future studies are clearly needed to reveal the descending facilitation modulation from ACC for migraine.

## STAR★Methods

### Key resources table

**Table:** 

REAGENT or RESOURCE	SOURCE	IDENTIFIER
**Antibodies**		

Rabbit antibody-GluN2A	Millipore	Cat#AB1555; RRID: AB_2112325
Rabbit antibody-GluN2B	Millipore	Cat#AB1557; RRID: AB_2112907
Rabbit antibody-GluR1	Millipore	Cat#AB1504; RRID: AB_2113602
Mouse antibody-GluR2	Millipore	Cat#MAB397; RRID: AB_2113875
Rabbit antibody-p-GluN2B-S1303	Millipore	Cat#07-398; RRID: AB_310582
Rabbit antibody-p-GluN2B-T1472	Millipore	Cat#AB5403; RRID: AB_177454
Rabbit antibody-p-GluR1-S831	Millipore	Cat#AB5847; RRID: AB_92077
Rabbit antibody-p-GluR1-S845	Millipore	Cat#AB5849; RRID: AB_92079
Rabbit antibody-AC1	Abcam	Cat#ab69597; RRID: AB_1267634
Rabbit antibody-GluN1	Millipore	Cat#05-432; RRID: AB_390129
Mouse antibody-Tubulin	Sigma-Aldrich	Cat#T5201; RRID: AB_609915
Gt Rb IgG(H+L)HRP	Millipore	Cat#AP307P; RRID: AB_92641
Gt Ms IgG(H+L)HRP	Millipore	Cat#AP308P; RRID: AB_92635

**Chemicals, peptides, and recombinant proteins**		

NB001	Sigma-Aldrich	SML0060; CAS: 686301-48-4
Bradykinin acetate salt	Sigma-Aldrich	B3259; CAS: 6846-03-3
Prostaglandin E2	Sigma-Aldrich	P5640; CAS: 363-24-6
Serotonin hydrochloride	Sigma-Aldrich	H9523; CAS: 153-98-0
Histamine	Sigma-Aldrich	V900396; CAS: 51-45-6

**Experimental models: Organisms/strains**		

Sprague Dawley male rats	Xi’an Jiaotong University, Xi’an, Shaanxi, China	RRID: MGI:5651135

**Software and algorithms**		

ORO	Ugo Basile	https://ugobasile.com/products/categories/pain-and-inflammation
SuperMaze	Xinruan	https://www.shxinruan.com/qxxw/
MED64 Mobius	Alpha	https://www.med64.com/med64support/software-downloads-support/
pClamp 10.3	Molecular Devices	RRID: SCR_011323
Minianalysis 6.0.3	Synaptosoft	RRID: SCR_002184
SoftMax Pro 6.2.1	Molecular Devices	RRID: SCR_014240
Image J	NIH	RRID: SCR_003070
Tanon MP	Tanon	http://biotanon.com/Product/ProductList
Origin 8.6	OriginLab	RRID: SCR_014212
Sigmaplot 10.0	Systat Software	RRID: SCR_003210
PASW Statistics 18	SPSS Inc.	RRID: SCR_002865

### Resource availability

#### Lead contact

Further information and requests for resources and reagents should be directed to and will be fulfilled by the lead contact, Min Zhuo (min.zhuo@utoronto.ca).

#### Materials availability

This study did not generate new unique reagents.

### Experimental model and subject details

#### Animals

Experiments were performed with adult male Sprague-Dawley rats (body weight 200-240 g). All rats used in this experiment were purchased from the Experimental Animal Center of Xi’an Jiaotong University and randomly housed under an artificial 12-h light/dark cycle (lights on 9 a.m. - 9 p.m.) with food and water provided *ad libitum*.

#### Institutional review board

Research protocols have been approved by the Ethics Committee of Xi’an Jiaotong University (approval IDs: No. 2017-714).

#### Surgery

Under isoflurane anesthesia, the rat was affixed in a stereotaxic frame and the scalp covering the dorsal surface was incised to expose the skull. A 1-mm diameter craniotomy was performed in the right frontal bone (1.5 mm lateral to midline and 1.5 mm posterior to the bregma) to expose the dura mater and a plastic cannula with a stainless-steel inner cannula was implanted into without touching the dura mater. The cannula was sealed with a matched obturator cap that had a tip just farther than the total length of the cannula, which prevented scar tissue from forming over the hole of the inner cannula. Sterile dental cement was applied around the cannula to protect and fix the cannula to the skull with the help of two small screws. The skin was sutured with 4-0 nylon, and only the obturator cap remained outside of the skin. Topical penicillin was applied after surgery to prevent infection of the surgical area and all rats received prophylactic antibiotic injections (penicillin, 0.1 million IU/100 g) for at least two days during recovery. The rat was returned to a clean individual cage when it was fully awake. All rats were allowed to recover for seven days before experiments. Periorbital sensory thresholds were measured during the recovery period to ensure that they returned to the pre-surgery baseline.

#### Treatment groups and repeated chemical stimulation

After post-surgical recovery, rats were randomly divided into the following three groups to receive repeated daily 10 μl injections of saline or IS on the dura mater for different numbers of days: (a) saline stimulation for one day (control group, Figure 1B upper), (b) IS stimulation for one day (acute headache (AH) group, Figure 1B middle), and (c) IS stimulation for seven days (migraine (M) group, Figure 1B lower). Furthermore, in order to observe the duration of migraine condition, the migraine group rats were tested respectively on the 1st day (M-1d), 2nd day (M-2d), 4th day (M-4d), 8th day (M-8d), 15th day (M-15d), 22nd day (M-22d) and 29th day (M-29d) of migraine (Figure 1C). The IS, a mixture of inflammatory mediators, consisted of 2 mM histamine, 2 mM serotonin, 2 mM bradykinin, and 0.2 mM prostaglandin E2 in saline at pH 7.4. Saline or IS was diffused around the dura mater while the rat was freely moving. All of these groups were tested at one hour after injection.

#### List of abbreviation

ACC: anterior cingulate cortex; ACSF: artificial cerebro-spinal fluid; AC1: adenylyl cyclase subtype 1; AMPA: α-amino-3-hydroxy-5-methyl-4-isoxazolepropionic acid; cAMP: cyclic adenosine monophosphate; CGRP: calcitonin gene-related peptide; EPM: elevated plus maze; EPSCs: excitatory postsynaptic currents; fEPSPs: field excitatory postsynaptic potentials; IS: inflammatory soup; LTP: long-term potentiation; mEPSCs: miniature excitatory postsynaptic currents; NMDA: N-methyl-D-aspartic acid; PAG: periaqueductal gray; PKMζ: protein kinase Mζ; TBS: theta-burst stimulation; TG: trigeminal ganglion.

### Method details

#### Orofacial operant test

Orofacial stimulation test system (31300, Ugo Basile) was used to test the mechanical withdrawal hyperalgesia. There were two standard rat cages in this test system, testing cage and companion cage. Rat initially was placed in the companion cage for 10 min to familiarize the environment. Subsequently, the rat was transferred to the testing cage and timed for 10 min to record its orofacial operant behaviors. In the anterior aspect of the testing cage, there was an apparatus with a drinking window for the rat head to enter and acquire a reward (milk) located on the opposing aspect of the drinking window. The apparatus also consisted of an infrared photo-beam and a detachable mechanical module containing 12 stimulating filaments. Depending on the type of the experiment, the detachable mechanical module was used or not to determine whether the rat was subjected to either no stimulus or mechanical stimulus when it attempted to poke its head through the drinking window. The infrared photo-beam located on the exterior aspect of the drinking window and was wired to a computer to automatically record the duration and the contact number of drinking milk. Before surgery, the rats underwent two weeks of pre-surgical adaptation training without mechanical stimulus. During the period of pre-surgical adaptation training, in order to reduce statistical error, drinking duration of more than 300 s was set up as a standard and the rats that couldn’t reach the standard would be eliminated. After one week of post-surgical recovery, the rats were performed two weeks of post-surgical adaptation training again without mechanical stimulus to ensure that the duration of drinking returned to the pre-surgical level. Similar to the adaptation training, although all experiments of different groups were performed with mechanical module, all of them were preceded by a 12-hour fasting period, 10 min for the rats to familiarize the testing environment, and a subsequent 10 min to allow for orofacial operant behavioral assessment.

#### Periorbital nociceptive threshold test

Periorbital nociceptive thresholds were tested by von Frey monofilaments (Ugo Basile). Von Frey monofilament was applied perpendicularly to the periorbital region until it buckled slightly, and it was held for 3 to 6 s or until a positive response was observed. The thresholds were determined by the ‘up-down’ method.^1^^,^^22^ A positive response was recorded when the rat withdrew its face from the von Frey monofilament. Rats that did not respond to the maximum filament strength (26 g) were assigned 26 g as their maximum periorbital nociceptive threshold for analysis.

#### Open field test

Open field test was designed to analyze anxiety-related behavior and locomotor activity. The apparatus comprised a circular black base (120-cm diameter) surrounded by black walls (40 cm). The inner space was a 90-cm diameter circle; the outside space was annular and 30-cm wide outside the inner space. Illumination was provided by a 40 W bulb. Animals were placed in the apparatus and allowed to explore freely for 5 min. Total distance and inner zone distance were recorded by an animal behavior trace analysis system (SuperMaze, Shanghai Xinruan). The apparatus was wiped with a 70 % alcohol solution between each test to remove olfactory cues.

#### Elevated plus maze (EPM) test

EPM was performed to measure anxiety-like responses and was conducted in a four-arm maze (10 cm× 50 cm) elevated 100 cm above the floor. The two closed arms had 40-cm high dark walls and the two open arms had 0.5-cm-high edges. The angle between the arms was 90°. Illumination was provided by a 40 W bulb. Rats were placed in the center of the apparatus facing a closed arm and allowed to explore freely for 5 min. Percent of time spent in the open arm and total number of arm entries were recorded by an animal behavior trace analysis system (SuperMaze, Shanghai Xinruan). The apparatus was wiped with a 70 % alcohol solution between each test to remove olfactory cues.

#### Microinjection of NB001 into the ACC

To determine the effects of synaptic plasticity in ACC in migraine, we performed local microinjection of NB001 into the ACC. Briefly, under isoflurane anesthesia, rats were placed in a stereotaxic instrument. Guide cannulas were implanted bilaterally above the ACC (2.0 mm anterior to bregma, 0.8 mm lateral from the midline, and 0.7 mm beneath the surface of the skull). Rats were given at least 2 weeks to recover after cannula implantation. On the 8th day of migraine attack (M-8d), injection cannulas that were 3 mm beneath the surface of the skull were used for intra-ACC injections. The microinjection apparatus consisted of a Gaoge glass syringe (1 μl). NB001 (10 mg/ml in saline) was infused for 10 min into each side of the ACC at a rate of 0.05 μl/min; an equivalent volume of saline was used as a control. After each injection, the microinjection needle was left in place for at least 2 min to prevent any solution flowing outward and behavioral tests were started at 30 min after injection.

#### Intraperitoneal injection and intragastric administration of NB001

NB001 was dissolved in saline and intraperitoneal injected in doses 5 or 20 mg/kg body weight, or intragastric administered in doses 60 mg/kg body weight on the 8th day of migraine attack (M-8d); an equivalent concentration of saline was used as a control. The effect of the drug was tested at 30 min after the injection.

#### Slice preparation

Acute coronal slices (300 μm) that contain the ACC were prepared from SD rats. Under 1-2 % isoflurane anesthesia, rat was sacrificed by decapitation. The entire brain was quickly removed from skull of the anesthetized rat and submerged in the ice-cold oxygenated (95 % O_2_ and 5 % CO_2_) cutting solution containing (in mM): 252 sucrose, 2.5 KCl, 6 MgSO_4_, 0.5 CaCl_2_, 25 NaHCO_3_, 1.2 NaH_2_PO_4_ and 10 glucose, pH 7.3-7.4. After brief cooling, coronal brain slices (300 μm) containing the ACC were cut by a Vibratome (VT1200S, Leica) in the above ice-cold oxygenated solution. Then, in a recovery chamber which was submerged with the artificial cerebrospinal fluid (ACSF) containing (in mM): 124 NaCl, 4.4 KCl, 2 CaCl_2_, 1 MgSO_4_, 25 NaHCO_3_, 1 NaH_2_PO_4,_ and 10 glucose, the coronal brain slices were incubated for at least 2 h at room temperature.

#### Preparation of the multielectrode array

The MED64 probe (P530A, Panasonic) that was used for extracellular field potential recordings in the experiments has an array of 64 planar microelectrodes, each arranged in an 8 × 8 pattern, with an interelectrode distance of 300 μm. To ensure the slice adhere to MED64 probe well during recording period, the new MED64 probe needs hydrophilic treatment. Before use, the surface of the MED64 probe was treated with 0.1 % polyethyleneimine (P-3143, Sigma-Aldrich) in 25 mM borate buffer (pH 8.4) overnight at room temperature. Before using the probe in the experiments, the surface of the probe was flushed at least three times with sterile distilled water to remove harmful substances that affect the activity of brain slices.

#### Extracellular field potential recording

After incubation in the recovery chamber, one coronal slice was transferred to the prepared MED64 probe and make sure the whole array of the microelectrodes covered the different layers of the ACC. In the recording chamber, the slice was further incubated for one hour under the condition that perfusion with oxygenated (95 % O_2_ and 5 % CO_2_) ACSF at 28-30 °C and maintained at a 2 ml/min flow rate. After one-hour incubation in the recording chamber, one channel located in the deep layer of ACC was selected as the stimulation site and biphasic constant-current pulse stimulation (0.2 ms) was applied to the stimulation site to evoke field excitatory postsynaptic potentials (fEPSPs). Before a theta-burst stimulation (TBS, five trains of bursts with four pulses at 100 Hz at 200 ms interval; repeated five times at intervals of 10 s) was applied on the stimulation site to induce LTP, the stable baseline responses were recorded for 30 min, and the fEPSPs responses were recorded for 3 h after LTP induction.

#### Whole-cell patch-clamp electrophysiology

Whole-cell patch clamp recordings were performed by Axon 200B amplifier (Molecular Devices), and a bipolar tungsten stimulating electrode was placed in deep layer of ACC to deliver the stimulations. For miniature EPSCs (mEPSCs) recording, neurons were voltage clamped at -70 mV in the presence of TTX (1 μM), and the recording pipettes (3-5 MΩ) were filled with a solution containing (in mM) 145 K-gluconate, 5 NaCl, 1 MgCl_2_, 0.2 EGTA, 10 HEPES, 2 Mg-ATP, 0.1 Na^3^-GTP (adjusted to pH 7.2 with KOH, 290 mOsmol). Meanwhile, the recording pipettes filled with a solution containing (in mM) 112 Cs-Gluconate, 5 TEA-Cl, 3.7 NaCl, 0.2 EGTA, 10 HEPES, 2 Mg-ATP, 0.1 Na^3^-GTP and 5 QX-314 (adjusted to PH 7.2 with CsOH, 290 mOsmol) were used for NMDA and AMPA receptor-mediated EPSCs recording. In the presence of AP-5 (50 μM), repetitive 0.05 Hz stimulations were applied to induced AMPA receptor-mediated EPSCs when neurons were voltage clamped at -70 mV. NMDA receptor-mediated EPSCs were recorded at -30 mV by bathing with CNQX (20 μM). Picrotoxin (100 μM) was always present to block GABA_A_ receptor-mediated inhibitory synaptic currents in all experiments. Clampex version 10.3 and Clampfit version 10.2 software (Molecular Devices) were used to collect and analyzed data.

#### Western blot analysis

Briefly, ACC samples were dissected on ice in cold PBS (-) and homogenized in lysis buffer (10 mM Tris-HCl (pH 7.4), 2 mM EDTA, 1 % SDS including a protease inhibitor cocktails). Samples were then centrifuged (15,000 g, 20 min, 4 °C) for supernatant. Sample protein concentrations were quantified using BCA assay (Beyotime), and electrophoresis of equal amounts of protein (40 μg) was performed on 7.5 % SDS-polyacrylamide gel. Separated proteins were transferred to polyvinylidene fluoride (PVDF) membranes, followed by blocking with 5 % skim milk in TBS-T (Tris-buffered saline with Triton X-100) at room temperature for 1 h, and were then probed with primary antibodies (Millipore, US): anti-GluN1 (1:500), anti-GluN2A (1:1000), anti-GluN2B (1:500), anti-p-GluN2B-serine 1303 (1:1000), anti-p-GluN2B-tyrosine 1472 (1:1000). Anti-GluA1 (1:500), anti-p-GluA1-serine 831 (1:1000), anti-p-GluA1-serine 845 (1:1000), anti-GluA2 (1:1000), anti-AC1 (1:1000), and anti-tubulin (1:4000; Sigma, US) at 4 °C overnight. The membranes were incubated with horseradish peroxidase-coupled anti-rabbit/mouse lgG secondary antibody diluted at 1:5000 (Millipore) for 1 h, followed by enhanced chemiluminescence detection of the proteins with Enhanced chemiluminescence, ECL (GE Healthcare). Membranes were used again for another primary antibody by stripping buffer (62.5 mM Tris-HCl (pH 6.8), 2 % SDS, 100 mM β-mercaptoethanol) followed blocking. Image J software (National Institute of Health) was used to assess the density of immunoblots.

### Quantification and statistical analysis

Data were expressed as mean ± SEM. In Figure 1, because behavioral data for different time points in the chronic migraine group were obtained from the same batch, and the control, AH, and chronic migraine groups were from different batches, we performed two-tail unpaired *t*-test to compare the differences of periorbital threshold and contact time between control and AH/chronic migraine groups. Moreover, we performed two-way ANOVA to compare the differences of cumulative contact time between control, AH, M-1d, and M-8d groups. In Figure 2, one-way ANOVA was used for statistical analysis because the rats were from different batches. For western blot and electrophysiological data, two-tail unpaired *t*-test, one-way and two-way ANOVA were used for statistical analysis because the rats were from different batches. In Figures 8, two-tail unpaired *t*-test was used for statistical analysis because the rats were from different batches. In Figure S1, two-tail paired *t*-test was used for statistical analysis because the rats before and after NB001 injection were from the same batch. In all cases, *p* < 0.05 was considered statistically significant.

## Data Availability

• All data produced in this study are included in the published article and its supplemental information, or are available from the lead contact upon request. • This paper does not report original code. • Any additional information required to reanalyze the data reported in this paper is available from the lead contact upon request.
