# Supplementary material for: Inhibiting neuronal AC1 for treating anxiety and headache in the animal model of migraine
Source: iScience. 2023 Apr 28;26(6):106790. doi: 10.1016/j.isci.2023.106790 (PMC10206497; doi:10.1016/j.isci.2023.106790)
Supplement: Document S1. Figure.S1 [file mmc1.pdf]

iScience, Volume *26*

Supplemental information

Inhibiting neuronal AC1 for treating anxiety and headache in the animal model of migraine

Ren-Hao Liu, Mingjie Zhang, Man Xue, Tao Wang, Jing-Shan Lu, Xu-Hui Li, Yu-Xin Chen, Kexin Fan, Wantong Shi, Si-Bo Zhou, Qi-Yu Chen, Li Kang, Qian Song, Shengyuan Yu, and Min Zhuo

Supplemental Figure 1

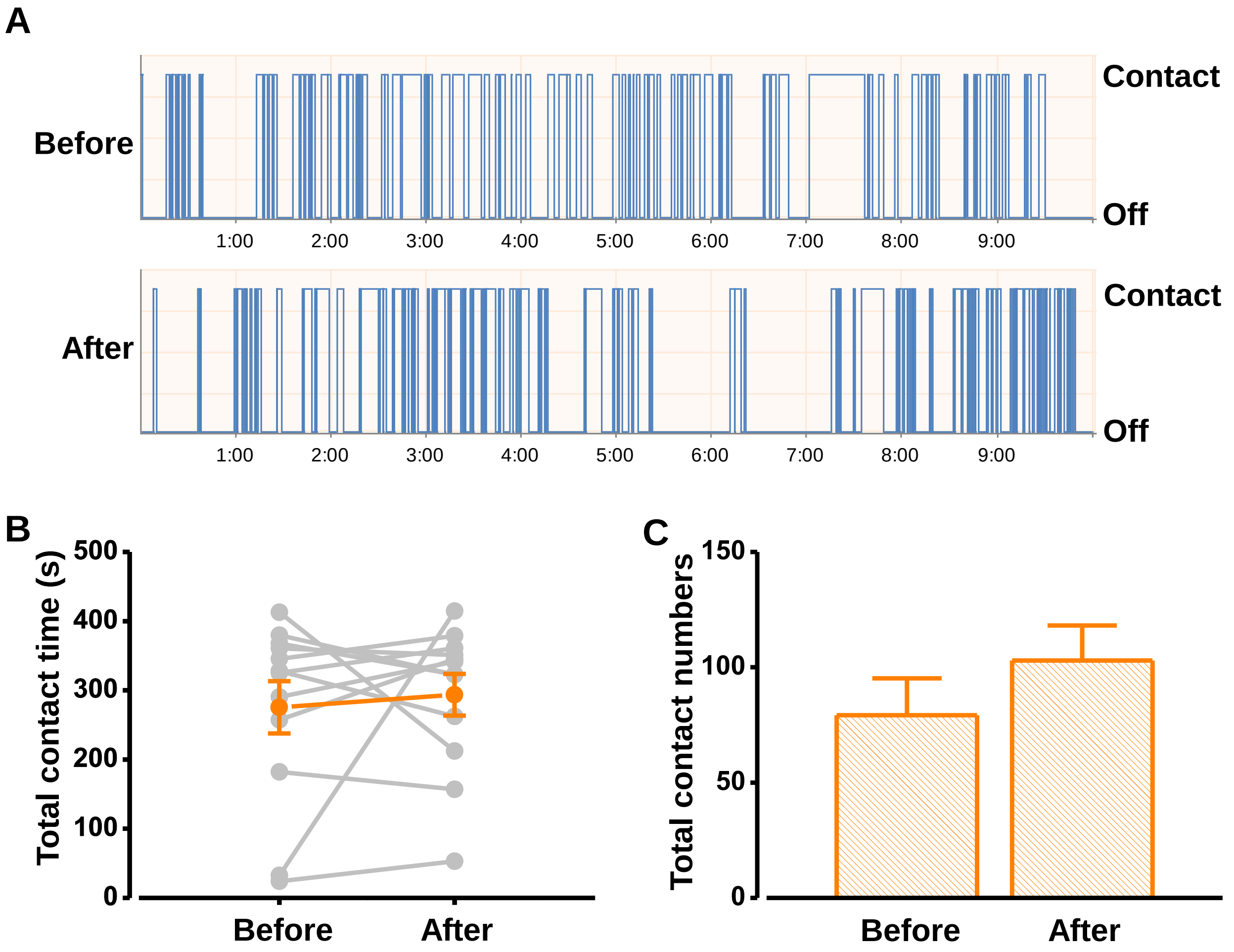

Figure S1. Low concentration NB001 didn't relieve periorbital hyperalgesia in migraine rats, related to Figure 7.

(A) Two sample traces show the automatic recordings of drinking behavior of M-8d rats in a duration of 10 min before (upper) and after (lower) NB001 injection.

(B) There was no difference in total contact time over the test period between before and after NB001 injection (n = 12 per group, $t_{(11)} = -0.458$, $p = 0.656$, paired *t* test).

(C) There was no difference in total contact numbers of M-8d rats before and after NB001 injection (n = 12 per group, $t_{(11)} = -1.865$, $p = 0.089$, paired *t* test).

Data are represented as mean ± SEM.
